# Interpretation of Strengthening Mechanism of Densified Wood from Supramolecular Structures

**DOI:** 10.3390/molecules27134167

**Published:** 2022-06-29

**Authors:** Kunpeng Li, Lihong Zhao, Junli Ren, Beihai He

**Affiliations:** State Key Laboratory of Pulp and Paper Engineering, South China University of Technology, Guangzhou 510640, China; felikp@mail.scut.edu.cn (K.L.); renjunli@scut.edu.cn (J.R.); ppebhhe@scut.edu.cn (B.H.)

**Keywords:** densified wood, strengthening mechanism, supramolecular structures, hydrogen bond, tensile strength, crystallinity

## Abstract

In this study, densified wood was prepared by hot pressing after partial lignin and hemicellulose were removed through alkaline solution cooking. The tensile strength and elastic modulus of densified wood were improved up to 398.5 MPa and 22.5 GPa as compared with the original wood, and the characterization of its supramolecular structures showed that the crystal plane spacing of the densified wood decreased, the crystallite size increased, and the maximum crystallinity (CI) of cellulose increased by 15.05%; outstandingly, the content of O(6)H⋯O(3′) intermolecular H-bonds increased by approximately one-fold at most. It was found that the intermolecular H-bond content was significantly positively correlated with the tensile strength and elastic modulus, and accordingly, their Pearson correlation coefficients were 0.952 (*p* < 0.01) and 0.822 (*p* < 0.05), respectively. This work provides a supramolecular explanation for the enhancement of tensile strength of densified wood.

## 1. Introduction

With environmental degradation and resource depletion, wood has attracted considerable attention from researchers due to its biodegradable, carbon-neutral, and inherently sustainable properties. Researchers have recently utilized and modified the intrinsic hierarchical structure of the wood to develop wood-based materials with specific properties, such as transparency [1,2], thermal management [3,4], electrical conductivity [5,6], and magnetism [7]. Most of these emerging applications reflected the lightweight and high-strength characteristics of wood-based materials. Accordingly, in-depth knowledge of the structure–strength relationship is the key to designing of wood-based materials with certain mechanical properties.

The skeleton component of wood-based materials is cellulose. Cellulose has excellent mechanical strength, such as cellulose nanofibrils with tensile strength up to 1.6 to 3.0 GPa [8] and crystalline cellulose I with the axial elastic modulus up to 130 to 150 GPa [9,10]. Presently, it is generally accepted that wood-based materials with higher cellulose content produce higher mechanical strength [11]. Therefore, partial removal of the matrix, especially the lignin, from wood allowed the preparation of high-strength, densified wood [2,12,13,14,15,16,17]. Specifically, wood was treated with a delignification process derived from the classical pulp production method while maintaining the aligned structure of wood fibers; subsequently, the delignified wood was densified by hot pressing.

The arrangement and stacking state of the cellulose molecular chains, defined as the supramolecular structures, is critical to the mechanical properties of densified wood. For instance, Jakob et al. [18] found that the tensile strength and elastic modulus decreased with the increasing tensile misalignment angle relative to fiber orientation and leveled off at misalignment angles of ≥30°. Khakalo et al. [15] used ionic liquids to convert the cellulose I of densified wood into cellulose II. They found that the tensile strength of densified wood decreased with the polymorphic transformation and the decreasing crystallinity.

The hydrogen bond was considered as the critical force determining the mechanical properties of cellulosic materials and densified wood [16,19,20,21,22]. Researchers have done much work on the relationship between them by simulations. For cellulose-based paper, Zhu et al. [20] showed that easier formation and reorganization of hydrogen bonds was crucial to simultaneously improving the strength and toughness of cellulose nanopaper. Meng et al. [23] developed a multi-scale crack bridging model. It suggested that bridging and hydrogen bonding between cellulose nanofibrils played an essential role in the toughening of cellulose nanopaper. In densified wood, cellulose molecular chains were in a highly aligned orientation, which amplified the enhancement of hydrogen bonding with the collective synergistic effect of molecular interlocking [24]. Molecular simulations by Song et al. [16] showed that hydrogen bonds could increase the sliding resistance between two adjacent lignocellulosic fibers of densified wood about 10-fold. In addition, the researchers have indirectly demonstrated the influence of hydrogen bonding on mechanical properties of cellulose-based paper and densified wood through experiments [25,26]. In the preparation of densified wood, Han et al. [26] investigated the effects of different drying methods and moisture content of delignified wood on the mechanical properties of the material and found a close relationship between them. There has been a lack of actual experimental data on the relationship between the tensile strength and hydrogen bonds of densified wood and cellulose-based materials.

In this work, densified wood with partially removed lignin and hemicellulose was prepared. The variations of intermolecular hydrogen bond content, crystalline structure, and crystallinity with cooking time were investigated. Based on the analysis of the supramolecular structures of cellulose, especially the content of intermolecular H-bonds, and the relationship between physical strength and the supramolecular structure, a molecular-level explanation for the enhanced tensile strength of densified wood was provided.

## 2. Results and Discussion

### 2.1. Tensile Strength and Morphology of Densified Wood

**Figure 1:**
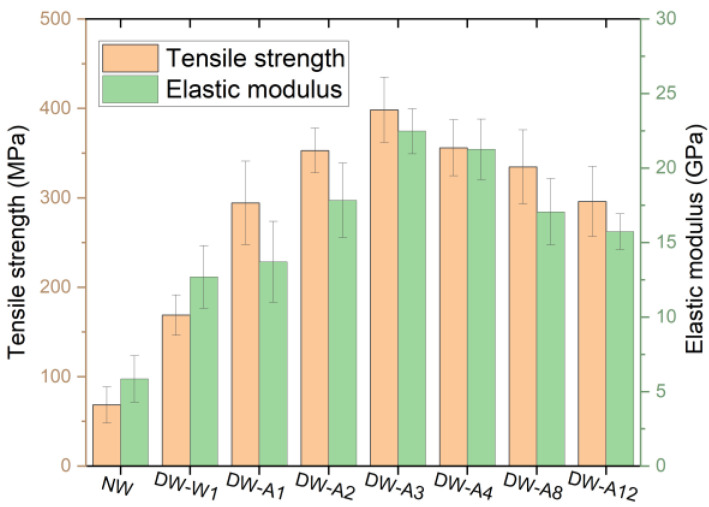
Tensile strength and elastic modulus of NW and densified wood.

To prepare densified wood, natural basswood (NW) was treated in boiled water or boiled NaOH and Na_2_SO_3_ aqueous solutions at different times following a hot-pressing process. There were two kinds of densified wood: densified wood treated with boiled water, named DW-W1, and densified wood treated with boiled alkali solutions for 1 h, 2 h, 3 h, 4 h, 8 h, and 12 h, named DW-A1, DW-A2, DW-A3, DW-A4, DW-A8, and DW-A12, respectively. As shown in Figure 1, Figure S1 and Table 1, the mechanical properties of densified wood were significantly improved compared with NW. The tensile strength and elastic modulus of DW-W1 increased to 168.9 MPa and 12.7 GPa, respectively. It was attributed to the densification that increased the stress-transfer capacity within fiber substrates. The tensile strength and elastic modulus of densified wood kept rising during the alkali cooking time of 1–3 h, and the maximum reached 398.5 MPa and 22.5 GPa for DW-A3, as seen in Table 1. They were a 4.8-fold increase in tensile strength and 2.8-fold in elastic modulus compared to NW. This phenomenon was generally considered as an increase in the intermolecular hydrogen bonding content due to the removal of lignin and hemicellulose [13,16]. With the further extension of alkali cooking time, the tensile strength declined, while in comparison with DW-A3, the tensile strength and elastic modulus for DW-A12 decreased by 102.3 MPa and 6.8 GPa, respectively.

**Figure 2:**
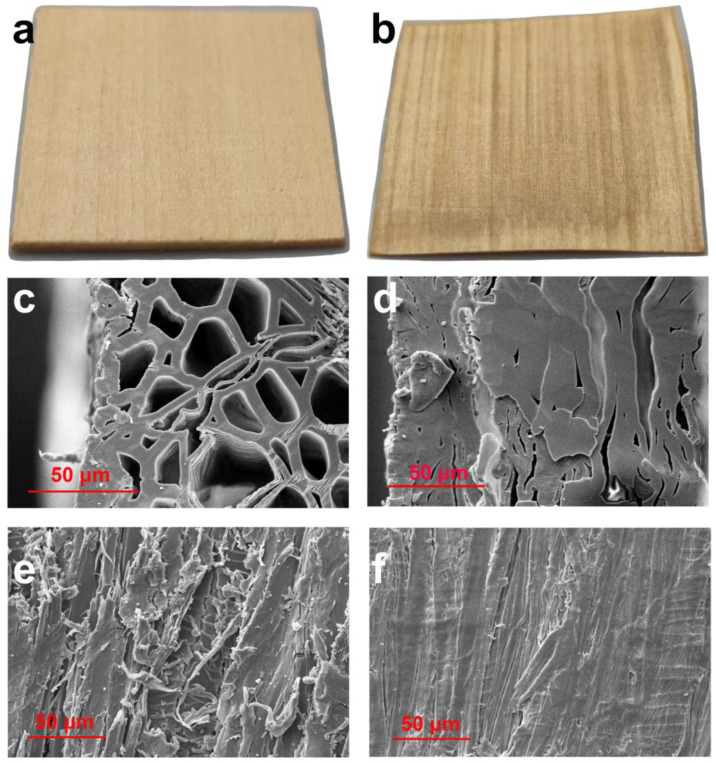
Photographs of (**a**) NW and (**b**) DW-A3 (densified wood from 3 h alkali cooking), cross-sections of (**c**) NW and (**d**) DW-A3, and longitudinal sections of (**e**) NW and (**f**) DW-A3.

As shown in Figure 2, compared with log NW in Figure 2a,c,e, the densified DW-A3 in Figure 2b,d,f had the following changes through alkali cooking and hot pressing. Compared to NW, the thickness of DW-A3 showed a reduction of approximately four times. Comparing Figure 2c,d, it can be seen that NW had many lumens with diameters around 10–20 μm, while the cell lumens and cell walls of DW-A3 collapsed and formed a tighter bond inside. Comparing Figure 2e,f, the gaps between fibers were diminished, the boundary between fibers was blurred, and the surface was denser and smoother.

### 2.2. Content of Intermolecular H-Bonds

**Figure 3:**
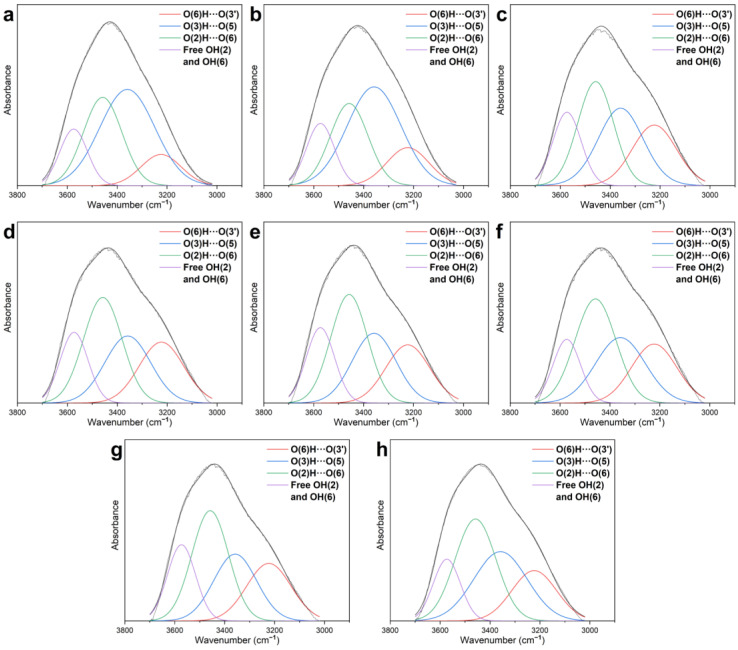
The deconvoluted FT-IR spectra of (**a**) NW and densified wood: (**b**) DW-W1, (**c**) DW-A1, (**d**) DW-A2, (**e**) DW-A3, (**f**) DW-A4, (**g**) DW-A8, and (**h**) DW-A12.

Taking the second derivative of the hydroxyl band from 3700 to 3000 cm^−1^ will improve the resolution of various H-bonds. In the second derivative spectrum, 3580–3550 cm^−1^ is assigned for free OH(2) and OH(6), 3455–3410 cm^−1^ is for O(2)H⋯O(6) intramolecular H-bonds, 3375–3340 cm^−1^ is for O(3)H⋯O(5) intramolecular H-bonds, and 3310–3230 cm^−1^ is for O(6)H⋯O(3′) intermolecular H-bonds. According to the peak positions of H-bonds and free hydroxyl groups, the bands of 3700–3000 cm^−1^ were deconvoluted, as in Figure 3. The relative contents of different hydrogen bonds and free hydroxyl groups were calculated according to the percentage of the area they occupied. The calculated results are shown in Table 2.

**Figure 4:**
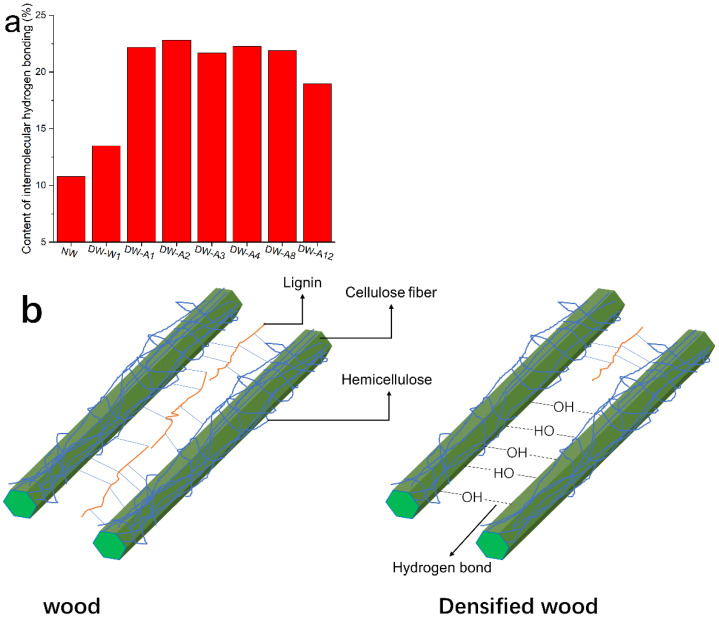
(**a**) The intermolecular H-bond content of NW and densified wood and (**b**) illustration of changes in cellulose interfibrillar components and the formation of intermolecular H-bonds in the wood through alkali cooking and densification.

As shown in Table 2 and Figure 4a, the content of O(6)H⋯O(3′) intermolecular H-bonds of DW-W1 cooked in water increased by 24.8%, from 10.8% to 13.5% compared to NW. Furthermore, the O(6)H⋯O(3′) H-bond content of each densified wood treated with alkali solutions was higher than DW-W1. This is because there is more lignin and hemicellulose removal by boiled alkali solution, allowing more intermolecular H-bond formation between the aligned fibers. Figure 4 illustrates the removal of lignin and hemicellulose and the formation of intermolecular H-bonds in densified wood treated with alkali solutions. O(6)H⋯O(3′) H-bond content within alkali cooking time ranging from 1 to 8 h was significantly higher than 12 h. The reduction of intermolecular H-bond content caused by the longer alkali cooking might be due to the destruction of H-bonds by OH^−^ in alkali [27,28]. The maximum content of O(6)H⋯O(3′) H-bond was observed in DW-A2, which increased by 111% from 10.8% to 22.8% compared with NW.

### 2.3. Crystal Structure and Crystallinity

**Figure 5:**
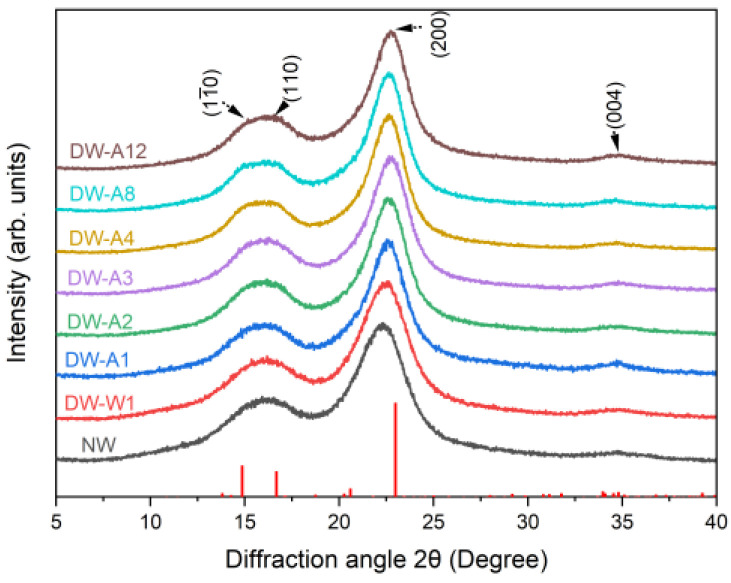
XRD diffractograms of the natural wood and densified wood.

As shown in Figure 5, the positions of the 11(_)0, 110, 200, and 004 lattice planes remained almost consistent for all samples, which indicated that the cellulose polymorph lattice shape did not change. Table 3 shows that the CI for DW-W1 increased by 5.02% compared to NW, which was consistent with the rising crystallinities after hot pressing in the literature [29,30]. It could be explained by co-crystallization [31]. The CI for densified wood showed an overall trend of first increasing and then decreasing with the alkali cooking time. It increased in the alkali cooking phase of 1–4 h, and compared with NW and DW-W1, the CI of DW-A4 increased by 15.05% and 10.03%, respectively. The rise in CI could be attributed to two factors. One was the increase of cellulose proportion with the removal of amorphous lignin and hemicellulose. Another was the co-crystallization of free cellulose chains adjacent to the crystalline region after lignin and hemicellulose removal [32,33]. The CI of densified wood slightly decreased as cooking time over 4 h, probably due to the peeling effect of alkali on cellulose crystal [34].

The value of interplanar spacing is related to the number of paracrystalline layers. It had a minimum between 0.384 and 0.3866 nm for perfect cellulose microcrystals [35,36]. As shown in Table 3, the interplanar spacing of all densified wood samples was smaller than that of NW. In particular, the reduction in the interplanar spacing was more significant for the alkali-cooked densified wood than the water-cooked one. It was consistent with the literature [37] and associated with the more intense stacking of cellulose chains after the partial removal of lignin and hemicellulose.

The crystallite size of DW-W1 was slightly reduced by 0.1 nm compared to NW. However, it significantly increased for the densified wood treated with alkali. DW-A4 and DW-A8 had the maximum crystallite size, both increasing by 0.9 nm compared to NW, which could be explained by co-crystallization. Compared with DW-A8, the crystallite size for DW-A12 was slightly reduced by 0.2 nm. It might be caused by the peeling action of the alkali.

### 2.4. Correlation Analysis

**Figure 6:**
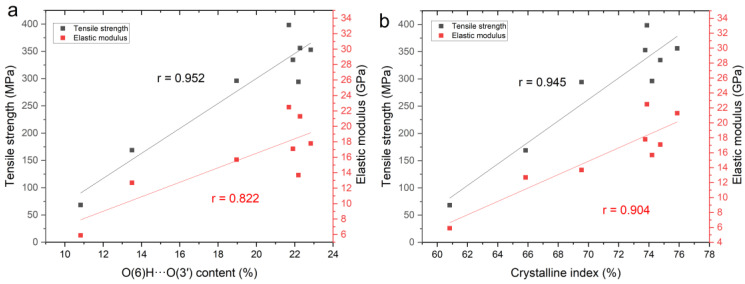
(**a**) Relationships between the content of intermolecular H-bonds and mechanical properties of natural and densified wood and (**b**) relationships between CI and mechanical properties of natural and densified wood.

Increasing the content of intermolecular H-bonds during cooking procedures resulted in a considerable increase in tensile strength and elastic modulus, as shown in Figure 6. The Pearson correlation coefficient between intermolecular H-bond content and tensile strength as well as between intermolecular H-bond content and elastic modulus was 0.952 (*p* < 0.01) and 0.822 (*p* < 0.05), respectively. It supports the molecular simulation results of Song et al. [16], which showed the pivotal contribution of intermolecular H-bonds to the enhanced strength of densified wood. Furthermore, the Pearson correlation coefficients between CI and tensile strength and between CI and elastic modulus were 0.945 (*p* < 0.01) and 0.904 (*p* < 0.01), respectively. The results illustrate the close relationships between the supramolecular structures of cellulose and the tensile properties of densified wood. Notably, some researchers considered that van der Waals forces also impacted the mechanical properties of cellulose materials [22,38,39]. Therefore, the correlation between tensile strength and density was also explored, and the Pearson correlation coefficient was 0.921 (*p* < 0.01), which was less than the 0.952 (*p* < 0.01) that was between tensile strength and intermolecular H-bond content. It might be due to the fact that H-bonds played a dominant role in the enhancing strength of densified wood, while van der Waals forces played a subordinate role.

## 3. Materials and Methods

### 3.1. Materials

Basswood, 30 × 30 × 1 mm; sodium hydroxide (AR, 96%); sodium sulfite (AR, 97%).

### 3.2. The Preparation of Densified Wood

**Figure 7:**
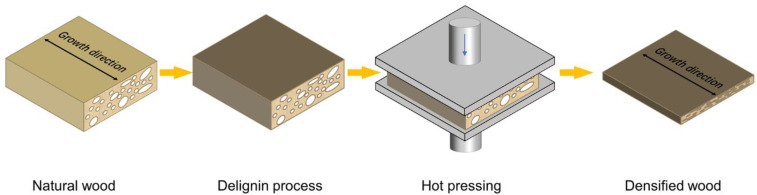
Schematic diagram of the preparation process of densified wood.

As shown in Figure 7, natural basswood veneers were cooked in boiled water for 1 h or boiled 2.5 M NaOH and 0.4 M Na_2_SO_3_ aqueous solutions (the liquid ratio was 20:1) for 1 h, 2 h, 3 h, 4 h, 8 h, and 12 h. Residual chemicals were removed with deionized water. The treated wood samples in the water-saturated state were covered with polytetrafluoroethylene plates and pressed with a hydraulic press under pressure of 2.5 MPa at 100 °C for 4 h to obtain densified wood.

### 3.3. Chemical Composition Analysis

The chemical compositions of the samples were analyzed according to the NREL protocol, including cellulose (glucan), hemicellulose (xylan, arabinose, galactan), and lignin (Klason lignin and acid-soluble lignin) [40].

### 3.4. Scanning Electron Microscope (SEM) Analysis

The microstructure of samples was characterized by the SEM (Zeiss, Merlin, Germany) with an acceleration voltage of 10 kV.

### 3.5. Mechanical Properties

The mechanical properties were carried out on an Instron 5565 universal tester (Boston, MA, USA) using a 2 KN load cell. The dry specimens were balanced in a standard conditioned room (23 ± 1 °C, 50 ± 2% RH) for 24 h before the test.

### 3.6. XRD Analysis

The crystalline structure was determined by a Shimadzu XRD 7000S (Shimadzu, Kyoto, Japan) while using Cu-Ka radiation. The samples were scanned at 40 kV and 30 mA in a 2θ range of 4–40° at 1° min^−1^. Before analysis, the experimental data were nine points smoothed with Savitzky–Golay algorithm. The crystalline index (CI) values were obtained from the peak intensity method. The CI was calculated as Equation (1) [41].

(1) $$\mathrm{CI}=\frac{{(I}_{200}{\;-\;I}_{m})}{I_{200}}\;\times \;100\%$$

where I_200_ is the maximum diffraction intensity value of the (200) lattice plane between the scattering angles of 2θ = 22° and 23°, and I_am_ is the minimum diffraction intensity of amorphous fraction between 2θ = 18° and 19°.

The crystallite size was calculated by the widely used Scherrer Equation (2) [42]:

(2) $$L=\frac{k\;\times \;\lambda }{\left(\beta \;\times \;\cos\theta \right)}$$

where L is the size that is perpendicular to the lattice plane, k is a constant that depends on the crystal shape (0.89), λ is the wavelength of the incident beam in the diffraction experiment (0.154 nm), β is the full width at half maximum of the diffraction peak, and θ is the Bragg’s angle.

### 3.7. FT-IR Analysis

The FT-IR spectra were recorded on solid samples in KBr pellets by means of an FT-IR Bruker Vertex 70 spectrometer (Karlsruhe, Germany). The spectra were recorded by averaging 64 recordings and at a resolution of 4 cm^−1^ with transmission mode from 4000 cm^−1^ to 400 cm^−1^. The results were made on the average spectra (seen in Figure S2) obtained from three recordings. Nonlinear fitting of the FT-IR spectral region from 3000 cm^−1^ to 3700 cm^−1^ was carried out with the Gaussian function [42].

## 4. Conclusions

In this work, densified wood with the enhanced strength was successfully prepared by cooking with hot water and alkali followed by hot pressing, and the relationship between the strength properties of densified wood and the supramolecular structures of cellulose was discussed. From the experimental results, alkali solution removed lignin and hemicellulose in wood fibers more efficiently than hot water, creating conditions for the formation of hydrogen bonds between cellulose molecules and thus more significantly improving the strength of densified wood. Finally, it was concluded that there was a significantly positive correlation between the tensile strength of densified wood and the supramolecular structures of cellulose, such as crystallinity and the intermolecular hydrogen bond content.

## Figures and Tables

**Table 1:** Chemical components, density, and tensile properties of NW and densified wood.

Samples	Lignin Content (%)	Cellulose Content (%)	Hemicellulose Content (%)	Density (g cm^−3^)	Tensile Strength (MPa)	Elastic Modulus (GPa)
NW	24.7 (0.4)	45.4 (0.5)	19.2 (0.3)	0.44(0.04)	68.4 (20.4)	5.9 (1.6)
DW-W1	24.7 (0.2)	45.3 (0.7)	19.5 (0.7)	1.06(0.05)	168.9 (22.6)	12.7 (2.1)
DW-A1	20.4 (0.1)	62.3 (1.2)	8.9 (1.2)	1.32(0.03)	294.3 (46.8)	13.7 (2.7)
DW-A2	20.2 (0.5)	64.7 (1.1)	9.3 (1.2)	1.31(0.00)	353.0 (25.0)	17.8 (2.5)
DW-A3	19.3 (0.6)	66.7 (0.9)	8.5 (0.9)	1.34(0.02)	398.5 (36.7)	22.5 (1.5)
DW-A4	18.4 (0.4)	68.7 (0.6)	9.7 (1.1)	1.33(0.02)	356.1 (31.2)	21.3 (2.0)
DW-A8	18.4 (0.7)	72.7 (0.8)	8.7 (0.5)	1.30(0.04)	334.7 (41.5)	17.1 (2.2)
DW-A12	19.0 (0.5)	74.3 (0.7)	8.0 (0.7)	1.26(0.02)	296.2 (39.3)	15.7 (1.2)

**Table 2:** The relative content of three H-bonds and the free OH of NW and densified wood.

Samples	O(6)H⋯O(3′)	O(3)H⋯O(5)	O(2)H⋯O(6)	Free OH(2) and OH(6)	R^2^
NW	10.8	46.1	29.6	13.5	0.9988
DW-W1	13.5	45.4	26.5	14.6	0.9988
DW-A1	22.2	29.5	31.1	17.2	0.9976
DW-A2	22.8	26.0	34.6	16.6	0.9976
DW-A3	21.7	26.1	34.2	18.0	0.9979
DW-A4	22.3	27.4	35.8	14.5	0.9976
DW-A8	21.9	24.8	35.0	18.3	0.9979
DW-A12	19.0	31.4	35.4	14.2	0.9984

R^2^ is the coefficient of determination.

**Table 3:** Crystalline index, crystallite size, and interplanar spacing of natural wood and densified wood.

Sample	CI (%)	Crystallite Size (nm) ^a^	Interplanar Spacing (nm) ^b^
NW	60.82	3.3	0.398
DW-W1	65.84	3.2	0.396
DW-A1	69.54	3.8	0.393
DW-A2	73.75	3.8	0.392
DW-A3	73.86	3.8	0.391
DW-A4	75.87	4.2	0.393
DW-A8	74.75	4.2	0.393
DW-A12	74.20	4.0	0.391

^a^ The size perpendicular to 200 planes; ^b^ the interplanar spacing of 200 planes.

## Data Availability

Not applicable.

## Supplementary Materials

The following supporting information can be downloaded at: https://www.mdpi.com/article/10.3390/molecules27134167/s1, Figure S1: stress-strain curve of NW and densified wood with various cooking time; Figure S2: FT-IR of NW and densified wood.
