# Supplementary material for: Interpretation of Strengthening Mechanism of Densified Wood from Supramolecular Structures
Source: Molecules. 2022 Jun 29;27(13):4167. doi: 10.3390/molecules27134167 (PMC9268594; doi:10.3390/molecules27134167)
Supplement: Supplementary file 1 [file molecules-27-04167-s001.zip › New Microsoft Word Document.pdf]

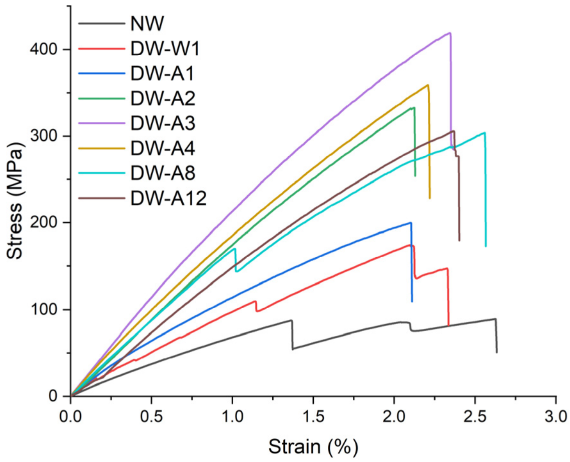

Figure S1. Stress-strain curve of NW and densified wood with various cooking time.

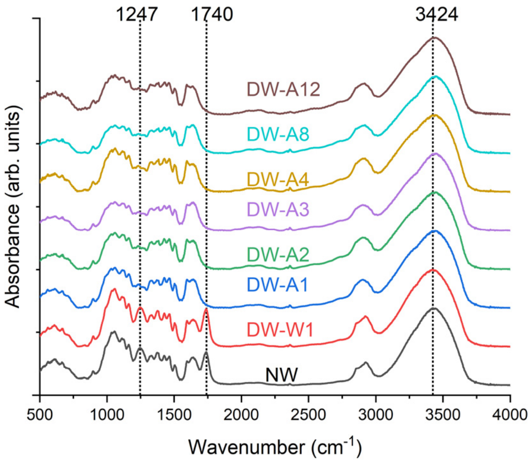

Figure S2. FT-IR of NW and densified wood.
